# Putative Auditory-Evoked Neurophonic Measurements Using a Novel Signal Processing Technique: A Pilot Case Study

**DOI:** 10.3389/fnins.2017.00472

**Published:** 2017-08-25

**Authors:** Alison M. Cook, Ashleigh J. Allsop, Greg A. O'Beirne

**Affiliations:** ^1^New Zealand Institute of Language Brain and Behaviour, University of Canterbury Christchurch, New Zealand; ^2^Eisdell Moore Centre Auckland, New Zealand

**Keywords:** cochlea, electrocochleography, cochlear microphonic, auditory neurophonic, hearing impairment

## Abstract

With changes to cochlear implant candidacy and improvements in surgical technique, there is a need for accurate intraoperative assessment of low-frequency hearing thresholds during cochlear implantation. In electrocochleography, onset compound action potentials (CAPs) typically allow estimation of auditory threshold for frequencies above 1 kHz, but they are less accurate at lower frequencies. Auditory nerve neurophonic (ANN) waveforms, on the other hand, may overcome this limitation by allowing phase-locked neural activity to be tracked during a prolonged low-frequency stimulus rather than just at its onset (Henry, 1995). Lichtenhan et al. (2013) have used their auditory nerve overlapped waveform (ANOW) technique to measure these potentials from the round windows of cats and guinea pigs, and reported that in guinea pigs these potentials originate in the cochlear apex for stimuli below 70 dB SPL (Lichtenhan et al., 2014). Human intraoperative round window neurophonic measurements have been reported by Choudhury et al. (2012). We have done the same in hearing impaired awake participants, and present here the results of a pilot study in which we recorded responses evoked by 360, 525, and 725 Hz tone bursts from the cochlear promontory of one participant. We also present a modification to the existing measurement technique which halves recording time, extracting the auditory neurophonic by recording a single averaged waveform, and then subtracting from it a 180° group-delayed version of itself, rather than using alternating condensation and rarefaction sound stimuli. We cannot conclude that the waveforms we measured were purely neural responses originating from the apex of the cochlea: as with all neurophonic measurement procedures, the neural responses of interest cannot be separated from higher harmonics of the cochlear microphonic without forward masking, regardless of electrode location, stimuli or post-processing algorithm. In conclusion, the extraction of putative neurophonic waveforms can easily be incorporated into existing electrocochleographic measurement paradigms, but at this stage such measurements should be interpreted with caution.

## Introduction

Over time, changes in the criteria for cochlear implant (CI) candidacy have led to growing numbers of CI candidates presenting with useable low-frequency (LF) hearing thresholds (i.e., <1 kHz). Improvements in minimally traumatic surgical techniques and the availability of “atraumatic” electrodes have improved the chances that this residual hearing may be preserved, enabling improved speech perception and appreciation of music (Gantz et al., 2005; Dorman and Gifford, 2010; Adunka et al., 2013). Intraoperative monitoring of LF hearing has the potential to help preserve this residual hearing (Mandalà et al., 2012). One approach has been to use cochlear response telemetry, using the CI electrodes themselves to monitor cochlear responses (Radeloff et al., 2012; Campbell et al., 2016). Of the cochlear potentials measurable using this technique, Campbell et al. have found that the onset compound action potential (CAP) and summating potential (SP) had poorer signal-to-noise ratios than the cochlear microphonic potential (CM), leading them to rely on the CM for intraoperative monitoring. While CM changes may indicate damage to the organ of Corti, the low-frequency CM amplitude recorded in the basal turn is not frequency specific (Patuzzi et al., 1989). It also does not provide information about the function of residual inner hair cells (IHCs) or neurons, and cannot be used for participants with non-functional outer hair cells (OHCs). Similarly, practitioners of electrocochleography (ECochG) have reported that while tone-burst stimuli allow estimation of auditory threshold for frequencies above 1 kHz, tone burst CAPs below 1 kHz are often smaller, because the slow onset/offset ramps required to avoid spectral splatter are less effective at eliciting synchronized neural firing at the onset of the tone burst, thereby underestimating LF sensitivity. Therefore, there is a need for a reliable intraoperative assay of very low frequency (<1 kHz) IHC/neural function in CI recipients.

One such assay may be the synchronized neural firing evoked *during* longer-duration LF tones. The cochlear response to ongoing tones has been measured since the earliest studies of cochlear potentials (Wever and Bray, 1930). Then, as now, a major issue was determining the source of the measured potential (i.e., cochlear or brain stem, OHC or neural). Because assumptions about generator sites are closely linked to the names given to such responses, nomenclature must be carefully considered. Over the decades, the response to ongoing tones has been given various names. In the earliest studies of cochlear potentials, the response termed the “Wever and Bray phenomenon” (Wever and Bray, 1930) in due course came to be understood as having both hair cell (cochlear microphonic) and neural contributions (Adrian, 1930; Adrian et al., 1931; Derbyshire and Davis, 1935). Similar responses measured with intra-cranial electrodes within various parts of the auditory brainstem were called “frequency following responses” (Boudreau and Tsuchitani, 1964; Worden and Marsh, 1968) but were later dubbed “auditory neurophonic” by Weinberger et al. (1970) to reflect their neural origin, and their similarity with the cochlear microphonic potential. Snyder and Schreiner (1984) reused this terminology but re-defined the “auditory neurophonic” as the response of individual auditory brainstem nuclei, and used the more specific term of “auditory nerve neurophonic” (ANN) to refer to the neurophonic measured differentially along the auditory nerve. Moreover, they reserved the (previously used) term “frequency-following response” to refer to activity measured from the scalp, which included auditory neurophonics from the auditory nerve, as well as higher auditory brainstem structures (Snyder and Schreiner, 1984, 1985). Henry (1995, 1997) and Choudhury et al. (2012) also used the term ANN, but this time referring to the neural component of the response measured from the round window (RW) of gerbils and humans, respectively. These authors used alternating condensation and rarefaction sound stimuli to cancel the first harmonic of the contributions to the averaged waveforms (assuming this to be dominated by the CM). This processing strategy cancels out the fundamental frequency of all response components, including the CM, leaving a smaller amplitude, frequency-doubled residual waveform containing the higher harmonics and baseline shifts of the hair cell and neural responses (Sellick et al., 2003). It is worth emphasizing that this frequency-doubling is a consequence of the summing of responses to alternating stimuli, and that any neural response in the *unprocessed* waveform will repeat at the stimulation frequency *f*, rather than at 2*f*. Lichtenhan et al. (2013) subsequently used the term the “auditory nerve overlapped waveform” (ANOW) to describe this same residual waveform recorded from the RW or nearby bone in cats and guinea pigs, albeit with the baseline shift removed to facilitate measurement of the AC component. Using a name other than “ANN” avoids the insinuation that the residual waveform is purely neural. However, the inclusion of “auditory nerve” in the “ANOW” name may also be problematic: any such waveform will inevitably contain both neural (ANN) and residual hair cell (CM) contributions, and it is not possible to determine the source of these higher harmonics by this processing strategy alone (see Section Discussion). In addition to “ANOW”, Lichtenhan et al. (2014) also used the term CR_ave, mid_ (i.e., the averaged cochlear response from the middle of the alternating tone burst) to acknowledge that multiple cochlear generators contribute to this response over a range of sound levels. In light of this ambiguity, here we will also refer to the response as CR_ave, mid_, or as the “putative neurophonic”.

We present here examples of the waveform recorded from the cochlear promontory in one participant (one ear). The invasive nature of the measurements limited our participant pool to subjects with suspected cochlear pathologies already undergoing transtympanic ECochG. We present in-depth results from one participant chosen for their clear tone-burst CAP responses and cochlear microphonic waveforms as seen in standard ECochG recordings, and use these (i) to demonstrate a novel technique that halves the averaging time for extracting steady-state tone responses and obviates the need for alternating condensation and rarefaction stimuli; (ii) to demonstrate that these measurements can be made as a relatively quick addition to any standard ECochG protocol; and (iii) to highlight the inherent ambiguity in any such waveform regarding contributions from the non-linear OHC receptor current (CM), and non-linear neural responses. This ambiguity is not an artifact of any particular processing algorithm, stimuli or electrode placement, but is intrinsic to the physiological mechanisms generating the CM and neurophonic. This point is critical, given the renewed clinical interest in the use of ECochG for intraoperative monitoring, and must be addressed before the relationship between neurophonic and audiometric thresholds can be established. It is not possible to confirm the neural origin of such a response without, for example, showing it is susceptible to forward masking (unlike hair cell responses), or by using neurotoxins such as tetrodotoxin or kainate, as is possible in experimental animals.

## Methods

### Patient selection and pre-testing

This study was carried out in accordance with the recommendations of the National Ethics Advisory Committee's “Ethical Guidelines for Intervention Studies”. The participant gave written informed consent in accordance with the Declaration of Helsinki. The protocol was approved by the Southern Health and Disability Ethics Committee (Ethics Ref: 14/STH/92). Following air- and bone-conduction audiometry and tympanometry, the participant underwent routine transtympanic ECochG in one ear only, as part of diagnosis for suspected Menière's syndrome (Allsop, 2016). In the end, for this participant the SP/CAP ratios in response to both clicks and tone bursts were not consistent with hydrops in the ear tested, according to Gibson's criteria (see Hornibrook et al., 2012). Audiometry revealed that the participant had a mild-to-moderate sensorineural hearing impairment in that ear: air conduction thresholds in dB HL (dB SPL in brackets) were 30 (55) at 250 Hz, 25 (35) at 500 Hz, 40 (45) at 1 kHz, 40 (50) at 2 kHz, 25 (35) at 4 kHz, 55 (70) at 6 kHz, and 60 (75) at 8 kHz, with bone conduction thresholds within 5 dB of air-conduction at the four frequencies tested (0.5, 1, 2, and 4 kHz). The contralateral ear showed a profound hearing loss, with responses unable to be measured at the limits of the audiometer.

### ECochG procedure

ECochG procedures used were the same as described in Hornibrook et al. (2012). The combined reference/ground electrode was placed on the forehead. Both electrodes were Ag/AgCl ECG electrodes (Blue Sensor; Ambu, Denmark). The tympanic membrane and ear canal were numbed with phenol before placing the monopolar transtympanic needle electrode (TECA; CareFusion, USA) onto the cochlear promontory. The electrode was held in place by a custom-made headphone holder, over which the magnetically shielded supra-aural headphone was placed.

Custom-written software was used to generate the stimuli, and record and process the responses. Tone burst stimuli at 360, 525, and 725 Hz (30 ms duration, 2 cycle rise-fall time) were presented at 18 stimuli/second at calibrated levels through the supra-aural headphone via a digital-to-analog converter (NI9269; National Instruments, TX, USA), and a battery-powered amplifier (MX28 MiniMix VI, Rolls Corporation). Sound stimuli frequencies were chosen to avoid harmonics of the 50 Hz mains power frequency. Where time constraints allowed (i.e., for 525 and 725 Hz), presentation levels were incremented in 5 dB steps, to obtain at least two responses above and below onset-CAP threshold. Sound levels are presented here as dB peSPL, which should allow the reader to reconstruct the stimuli used in this study. While we did not measure psychophysical detection thresholds to these stimuli, we assume they would lie between those recorded by Poulsen and Legarth (2008) for 5 ms tone bursts, and the long-duration tones used in audiometry (ANSI, 2004).

The ECochG response was amplified with an electrically isolated bioamplifier (MK15; Amplaid, Milan, Italy), band-pass filtered at 0.5 Hz and 3 kHz (1st order high-pass, 2nd order low-pass), and sampled at 44.1 kHz (NI9222; National Instruments, TX, USA). Averaging and processing of the responses was performed by our software. Whole averaged ECochG waveforms (*n* = 300–310) were recorded, and the plateau region of the response was used for post-processing.

The analysis window was chosen to be during the plateau (*after* the tone burst onset CAP), where the amplitude of the response has largely adapted. The exact analysis window varied with frequency, commencing 1.5 stimulus cycles after the onset CAP at response threshold, and included an integer number of stimulus cycles (4 cycles for 360 Hz, 8 for 525 Hz, and 13 for 725 Hz) before the start of the stimulus offset ramp. The noise floor and pre-stimulus DC offset were calculated from the 5 ms pre-stimulus window. The entire averaging process lasted ~10 min per ear when presenting alternating stimuli at three frequencies and six sound levels.

**Figure 1:**
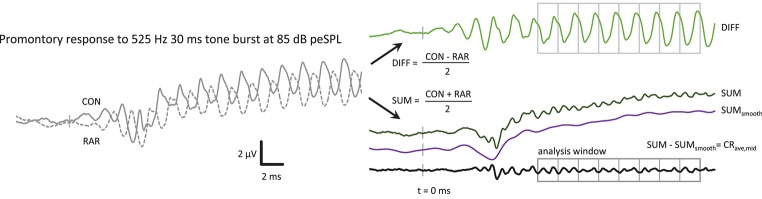
The post-processing steps to produce the CR_ave,mid_ from the ECochG response, in this case a 525 Hz 85 dB peSPL tone burst. CON and RAR are the averaged ECochG responses to condensation and rarefaction tone bursts, respectively. The CR_ave,mid_ is the sum of the CON and RAR responses, with the baseline shift removed. The SUM_smooth_ waveform was produced by low-pass filtering the SUM waveform at *f*. The analysis window was further divided into epochs with a duration of one cycle of the stimulus in length for further averaging of the CR_ave,mid_. The averaged CR_ave,mid_ waveform therefore contains 2 cycles of CR_ave,mid_ at 2*f*.

Responses from condensation (“CON”) and rarefaction (“RAR”) tone bursts were averaged separately. After removing any DC offset, the CON and RAR waveforms were summed and divided by 2 to produce the “SUM” waveform (see Figure 1) with the aim of canceling, or at least minimizing, any contributions that are of opposite polarities in the CON and RAR responses (assumed to be dominated by CM). The RAR waveform was subtracted from the CON waveform, and the result divided by 2 to produce the “DIFF” waveform, which allowed examination of the putative CM contribution.

The SUM waveform contained a slow baseline shift, which could be removed by subtracting a bandpass filtered version of the SUM response (high-pass at 0.01 Hz, low-pass at stimulus frequency, both with 35 dB/octave roll-off) from the unfiltered SUM waveform, leaving the CR_ave,mid_ waveform that is the focus of this study.

### Averaging within the tone burst

As in Lichtenhan et al. (2013), the signal-to-noise ratio of the waveform could be further improved by dividing the analysis region of the CR_ave,mid_ waveform into epochs the length of one cycle of the stimulus frequency *f* (or two cycles of the 2*f* CR_ave,mid_). These epochs were then averaged together (Figure 1). For the 360 Hz tone burst, 4 stimulus cycles were averaged, increasing the SNR by 6 dB (√4) or reducing the time taken to reach a given SNR by 4-fold. Similarly, averaging time was reduced at 525 Hz and 725 Hz by 8-fold and 13-fold, respectively, with increases in SNR of 9 dB and 11 dB, respectively.

### Sham control responses

**Figure 2:**
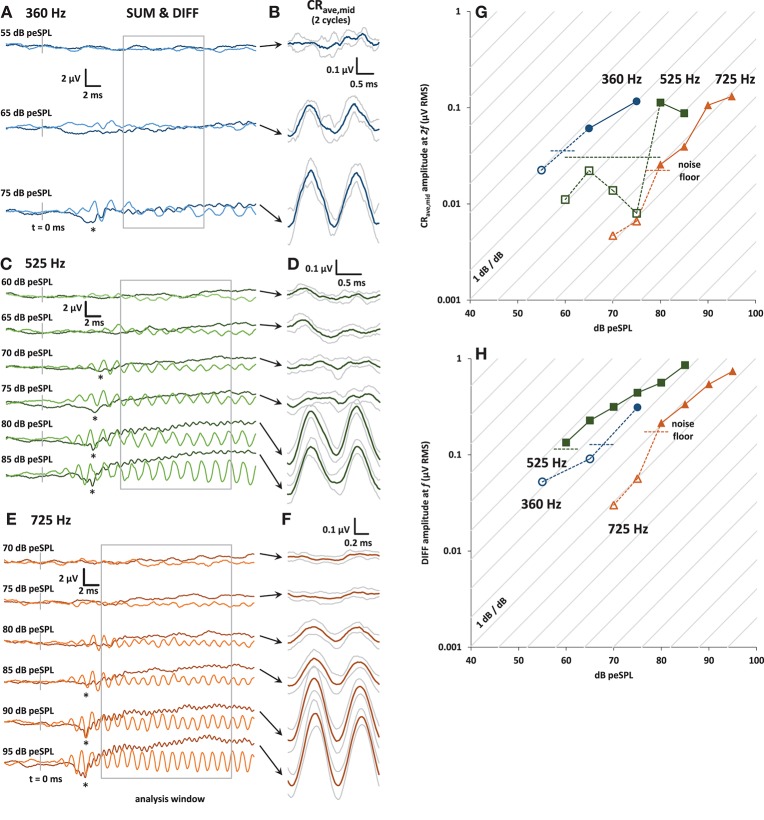
**(A)** Plots of averaged SUM (dark trace) and DIFF (light trace) responses to 30 ms condensation and rarefaction tone bursts at 360 Hz. The SUM trace is equivalent to averaged ECochG responses to alternating stimuli. The onset CAP can be seen in the SUM trace at the highest level presented (asterisk). The analysis window is shown in gray. **(B)** CR_ave,mid_ waveforms (±1 s.d.) obtained by further averaging of the baseline-shifted SUM waveforms shown in **(A)**. The analysis window was divided into integer multiples of the stimulus cycle at *f*, and so contains 2 cycles of the CR_ave,mid_ at 2*f*_._**(C)** and **(E)**: As for **(A)**, with tone bursts at 525 and 725 Hz, respectively. **(D)** and **(F)**: As for **(B)**, with tone bursts at 525 and 725 Hz, respectively. **(G)** Input-output curves for the CR_ave,mid_ response amplitude, calculated from the amplitude of the 2*f* spectral peak of the baseline shifted SUM waveforms at 360 Hz (blue circles), 525 Hz (green squares), and 725 Hz (orange triangles). The noise floor (horizontal dashed lines) was calculated from the RMS amplitude of the waveforms in the 5 ms pre-stimulus window for each frequency and stimulus presentation level, and then averaged to produce the average noise floor value shown for each frequency. CR_ave,mid_ amplitudes that are below the noise floor are shown with open symbols and dotted lines. **(H)** Input-output curves for the DIFF response amplitude, calculated from the amplitude of its spectral peak at *f*.

As in any electrophysiological response that follows the sound stimulus, it is essential to confirm that the recorded responses are not the result of electromagnetic feed through between the headphone and the recording electrode. If using insert earphones, control responses could be obtained simply by clamping off the sound delivery tube or blocking the ear canal, but this was not possible with the supra-aural headphones used in this study, with an electrode placed through the tympanic membrane. This is a limitation of this study. However, as shown in Figure 2, the CR_ave,mid_ and DIFF responses did not grow with sound level by 1dB/dB (gray lines in Panels G and H), as would be expected from electrical capacitive feedthrough from the headphone transducer. Moreover, our focus on the higher harmonics of the averaged responses makes any residual linear feed-through of little concern.

## Results

Figure 1 shows an example of the sequence of post-processing steps to produce a CR_ave,mid_ waveform. The CR_ave,mid_ waveform is essentially the sum of the condensation and rarefaction tone burst responses, with the baseline shift removed to facilitate processing (i.e., further averaging within the plateau region analysis window). Note that the CR_ave,mid_ appears as a frequency-doubled waveform (i.e., at 2*f*) as a result of the summing of condensation and rarefaction stimuli. The putative neurophonic appears in the CON and RAR waveforms at *f*, where it contributes to their distorted wave shapes (Figure 1).

In Figure 2 panels A, C, and E are plots of the entire 30 ms- long SUM and DIFF waveforms over a range of stimulus sound levels. The CAP at the tone-burst onset is visible in the SUM waveform (indicated by an asterisk in Panels A, C, and E). The SUM waveform is equivalent to the averaged response from alternating stimuli commonly used in ECochG. The decrease in CAP latency with increasing stimulus sound level can also be clearly seen for 525 and 725 Hz. Unfortunately, due to time constraints, not all sound stimulus levels were tested at 360 Hz. Panels B, D, and F of Figure 2 show the corresponding CR_ave,mid_ waveforms for each sound level, obtained as shown in Figure 1. The gray traces above and below these averaged CR_ave,mid_ waveforms (shown in black) represent ± 1 standard deviation (calculated across the number of averaged stimulus cycles in the analysis window; i.e., *n* = 4, 8, and 13 for 360, 525, and 725 Hz, respectively).

Panels G and H of Figure 2 show input-output functions for the CR_ave,mid_ and DIFF. The amplitude values of CR_ave,mid_ and DIFF were obtained from the spectrum at 2*f* and *f*, respectively. Responses below the noise floor are shown with open symbols. The noise floor for visual detection for each input/output function was calculated as the mean RMS amplitude of the averaged trace in the pre-stimulus window (5 ms before tone-burst onset).

The growth of the CR_ave,mid_ and DIFF responses out of the noise floor shown in the input-output functions can be seen in the averaged traces (Figures 2A,C,E). The diagonal lines in panels G and H of Figure 2 represent the 1 dB/dB growth expected for a capacitive feed-through electrical artifact.

### An alternative processing strategy

**Figure 3:**
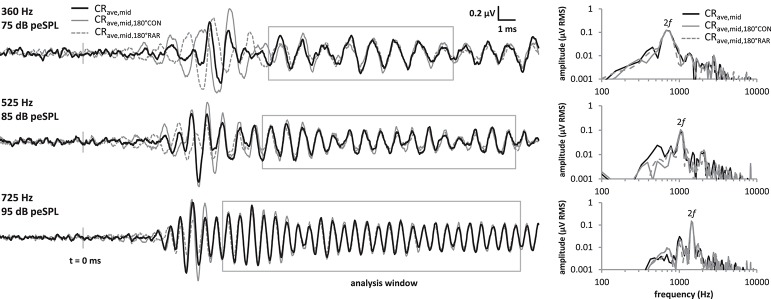
Comparison of the CR_ave,mid_ processing strategies at the 3 frequencies tested. Responses were obtained by averaging CON and RAR alternating stimuli as in standard ECochG (“CR_ave,mid_”, black solid traces), and also by presenting only CON tonebursts, and using a 180° group-delayed version of CON response to replace the RAR responses during the processing (“${\text{CR}}_{\text{ave},\text{mid},180^{{}^{\circ}}CON}$”, gray solid traces). Similarly, the “${\text{CR}}_{\text{ave},\text{mid},180^{{}^{\circ}}RAR}$” responses (gray dotted traces) were obtained by presenting only RAR tonebursts and using a 180° group-delayed version of the RAR response to replace the CON response during the processing. Although, the onset CAPs differ in latency for these three methods, these fall outside our CR_ave,mid_ analysis window. Within the analysis window (gray boxes), the CR_ave,mid_, ${\text{CR}}_{\text{ave},\text{mid},180^{{}^{\circ}}CON}$ and ${\text{CR}}_{\text{ave},\text{mid},180^{{}^{\circ}}RAR}$ waveforms mostly overlie. Similarly, the spectra of the three waveforms (right) within these analysis windows also overlie.

Because the analysis time window covered a relatively stable region of the LF-evoked promontory response waveform and excluded any onset components, we were able to employ a novel variation of the technique described above that halved the time taken to obtain an averaged response. This was achieved by presenting only CON tone bursts, and using a 180° group-delayed version of the CON response to replace the RAR responses during the processing described above, producing the trace shown as the ${\text{CR}}_{\text{ave},\text{mid},180^{{}^{\circ}}CON}$ waveform in Figure 3. Similarly, if only rarefaction tone bursts were presented then group-delayed RAR responses could be used instead of CON responses (${\text{CR}}_{\text{ave},\text{mid},180^{{}^{\circ}}RAR}$ in Figure 3). In both cases, the exact delay applied corresponded to half of one cycle of the stimulus frequency. These three processing methods are compared in Figure 3, both in the time and frequency domains.

The three waveforms do not overlie at the beginning of the tone burst, because the transient onset components differ in latency between condensation and rarefaction responses (Peake and Kiang, 1962). However, the onset-CAP falls outside the analysis window used in our and previous studies. Within the analysis window the three waveforms mostly overlie, as do their amplitude spectra calculated over this same window.

## Discussion

The results of the present study have been obtained using variations on the methods and post-processing strategies described by Henry (1995, 1997), Adrian (1930), and Lichtenhan et al. (2013, 2014). The novel averaging strategy presented here halved the averaging time without substantially changing the response for this participant (Figure 3), and within-tone-burst averaging improved the signal-to-noise ratio by a factor proportional to the number of analyzed cycles. Ideally, the length of these tone bursts could be greatly increased, thereby lengthening the usable analysis window and further improving the signal-to-noise ratio. This measure would further reduce the averaging time if using a fixed SNR criterion for response detection. It would also improve the frequency specificity of the stimulus by reducing spectral splatter often present in short-duration tone-bursts. These advantages may outweigh the reduction in response amplitude that may result from excluding the pre-plateau components from the analysis window.

We and others are interested in the neurophonic waveform as an objective indicator of low-frequency cochlear sensitivity that can be added to existing ECochG protocols. The waveform may be of particular use for i) objective measurement of low-frequency thresholds/cochlear function in the clinic, and ii) intraoperative monitoring during ear surgery for patients with serviceable low-frequency hearing (e.g., CI recipients). CM recordings during implantation may prove a useful indicator of generalized damage to the organ of Corti (Campbell et al., 2016), and may also provide information regarding OHC operating point shifts caused by cochlear pressure and fluid balance changes (Patuzzi and Moleirinho, 1998). However, the CM is an assay of local OHC function only; a reliable frequency-specific assay of cochlear nerve sensitivity would be useful.

Unfortunately, we cannot conclude that the CR_ave,mid_ waveforms presented here were purely neural, nor that they originated solely from the cochlear apex. This is because (a) no post-processing strategy can distinguish between cochlear microphonic and neurophonic, because the two responses will have varying degrees of both symmetric and asymmetric distortion, depending on sound level and pathology; (b) no additional procedure to assess the neural component (e.g., forward masking) was performed; and (c) our participant did not have normal hearing. The last point means we cannot rely on evidence from previous studies illustrating the reliability of the CR_ave,mid_ as a measure of neural function for stimuli presented below certain sound levels.

The issue of the separation of CM and neurophonic is not new (see, for example, Marsh et al., 1970; Snyder and Schreiner, 1984; Chimento and Schreiner, 1990; Forgues et al., 2014), and must be considered in any future studies of neurophonic waveforms, because the neurophonic and the CM occur concomitantly in cochlear recordings to varying degrees depending on recording location, electrode montage and pathology. Even for differential recordings along the cochlear nerve at the internal auditory meatus (e.g., Snyder and Schreiner, 1984), the CM may be present to a degree because of the proximity of the electrode locations to the cochlear fluids (see Stegeman et al., 1997, Pastras, under review).

Ideally then, to improve the reliability of the CR_ave,mid_ as an estimate of low-frequency sensitivity of the cochlear nerve, recordings should be performed with an electrode placement or montage that limits the contribution of cochlear hair cell potentials and maximizes the contribution of the cochlear nerve electrical activity. For example, we would expect that placing the non-inverting electrode on the promontory rather than the RW would reduce the amplitude of the CM, with little attenuation of the neurophonic. This assumes that the neurophonic, like the CAP, is a field-potential whose dipole is localized to the internal auditory meatus (Brown and Patuzzi, 2010; Rattay and Danner, 2014), whereas the CM is a field potential whose dipole spans the basilar membrane, and which electrically partially cancels at locations such as the bony regions of the middle ear. That is, by utilizing differences in the electrotonic spread of the VIIIth nerve field potential and cochlear hair cell field potential, it should be possible to choose a recording location that has an optimal nerve:hair cell contribution, in regards to their electrical activity. We have not compared recording locations in this study, and we do not suggest that the promontory is by any means the optimal recording location for neurophonic potentials, but the promontory should have a better neural:CM ratio than the RW. This issue should be considered in future measurements, because any reduction in the hair cell component of the response would reduce averaging time and increase certainty about the neural threshold, both of which are crucial considerations for real-time intraoperative monitoring of peripheral sensitivity. It is important to note that optimal electrode recording location will reduce but not eliminate possible “contamination” of neural responses by CM.

### Methods for separating hair cell and neural contributions

Averaging of responses to alternating polarity stimuli is routinely used in ECochG and provides “good enough” cancelation of CM for detection of onset-CAP. However, it will not cancel the CM unless the CM waveform is symmetric. It has been proposed that CM and neural components could be separated using spectral analysis of the CR_ave,mid_ waveform, assuming asymmetric distortion of CM and half-wave rectification of neural responses (Choudhury et al., 2012; Forgues et al., 2014). This method is unreliable, however, because the CM can distort symmetrically *or* asymmetrically, depending on the operating point of the non-linear transfer curve relating the opening probability of the mechanoelectrical transduction channels and the flow of current into the OHCs (Patuzzi and Moleirinho, 1998). Furthermore, OHC operating point is labile, particularly as a result of exposure to (intense) low-frequency tones (O'Beirne, 2005) or as a result of cochlear pathology such as Menière's syndrome or endolymphatic hydrops (Sirjani et al., 2004; Brown et al., 2013). Similarly, neural response phase varies with sound level (e.g., “peak-splitting”; Kiang, 1990) and following acoustic trauma (Patuzzi and Sellick, 1983). Thus, it is not possible to isolate the underlying cause of changes in the magnitude or phase of spectral components in any given participant, without application of additional measurement techniques, or *a priori* knowledge of the underlying physiology. In animal experiments, Henry (1995, 1997) and Lichtenhan et al. (2014) used tetrodotoxin to block neural responses and reported that, at least in their experiments, a significant proportion of the response measured at the RW was neural in origin. Nevertheless, the question remains whether the source of a response obtained with a human participant in a clinical setting is predominantly neural or OHC, particularly because any given participant will have their own individual pattern of OHC and/or neural hearing loss.

Forward masking presents one potentially useful clinical method of separating CM and neurophonic. Henry (1995, 1997) has demonstrated the use of forward masking of neural responses in RW measurements to obtain “pure” CM waveforms that could be subtracted from the raw waveform to produce a “pure” ANN. This process is analogous to the masking protocol presented by Chimento and Schreiner (1990) for removing CM from scalp-recorded FFR, and has the advantage that the resultant waveform retains the large amplitude response at the stimulus frequency (Chimento and Schreiner, 1990), unlike the summing of responses to alternating polarity stimuli.

### CR_ave,mid_ and audiometric thresholds

We were not able to compare audiometric thresholds to CR_ave,mid_ threshold here, because (i) the CR_ave,mid_ was obtained at non-standard frequencies (for which audiometric thresholds were not measured) in order to avoid harmonics of 50 Hz mains interference, and (ii) because of the limited amount of data obtained (3 frequencies only). Approximate audiometric thresholds at 360, 525, and 725 Hz (obtained by interpolating from the audiogram data—see Section Methods) did not show a clear relationship to CR_ave,mid_ thresholds, nor did onset-CAP thresholds obtained from the SUM waveforms in Figure 2. Audiometric thresholds, together with onset-CAP and CR_ave,mid_ input-output functions should be obtained in a large number of both normal and hearing-impaired participants to determine the relationship between CR_ave,mid_ and audiometric threshold.

### Neurophonic frequency specificity

Another issue that must be considered in interpreting CR_ave,mid_ amplitudes is the basal-ward recruitment of neural firing at high sound levels (Snyder and Schreiner, 1985). CR_ave,mid_ measurements in (normal hearing) guinea pigs show a significant neural component originating in the cochlear apex only for sound levels of 70 dB SPL or less (Lichtenhan et al., 2014). This issue is further complicated for individuals with hearing loss: the low-frequency tuning curve tails of high characteristic frequency neurons can become hypersensitive with particular patterns of neural/inner hair cell and OHC damage (Liberman and Dodds, 1984; also reviewed in Patuzzi and Robertson, 1988). That tail hypersensitivity also occurs with temporary threshold shift after acoustic trauma (Patuzzi and Sellick, 1983) is a salient point if measuring neurophonic responses intraoperatively before and after temporal bone drilling. High characteristic frequency neuron tail responses could be reduced by masking.

## Conclusions

Incorporating neurophonic measurement into standard ECochG protocols may offer an attractive method for objectively estimating the sensitivity of the apical portions of the cochlea. However, the fact that the CM *and* neurophonic can have varying degrees of both symmetric and asymmetric distortion in any given participant means that no post-processing algorithm can reliably separate these two components (either in the time- or frequency-domains). Before the relationship between the neurophonic and audiometric threshold can be established in normal hearing and pathological ears, future research in humans must determine optimal electrode montages that reduce CM contamination of neurophonic responses at the “front-end”, and most importantly, pursue masking techniques that ensure reliable separation of neural and hair cell responses, and which increase the frequency selectivity of the measured neurophonic waveform. These issues must be addressed in a timely manner given the growing interest in the use of the neurophonic as an objective measure of low-frequency cochlear function.

## Author contributions

This study was designed by GO. Measurements were made by GO and AA with assistance from Jeremy Hornibrook and Gurjoat Vraich. GO, AA, and AC conducted data analysis. AC and GO wrote the manuscript.

### Conflict of interest statement

The authors declare that the research was conducted in the absence of any commercial or financial relationships that could be construed as a potential conflict of interest.
